# The Body Mass Index-Mortality Link across the Life Course: Two Selection Biases and Their Effects

**DOI:** 10.1371/journal.pone.0148178

**Published:** 2016-02-03

**Authors:** Hui Zheng, Jonathan Dirlam

**Affiliations:** Sociology Department, Institute for Population Research, Ohio State University, Columbus, Ohio, United States of America; Tulane School of Public Health and Tropical Medicine, UNITED STATES

## Abstract

In this study, we investigated two selection biases that may affect the obesity-mortality link over the life course: mortality selection and healthy participant effects. If these selection mechanisms are stronger among obese adults than among non-obese adults, they may contribute to the weakening obesity-mortality link over the life course. We used data from the National Health and Nutrition Examination Survey 1988–2010 with linked mortality files from 1988–2011. We employed weighted Cox models to test and adjust for these two selection biases. We also used complementary log-log models, adjusted for a normal distribution of frailty, to test for mortality selection effects; accelerated failure-time models to mitigate the mortality selection effect; and ordinary least squares regression to test for healthy participant effects. The link between class II/III obesity and mortality weakens at older ages. We did not find evidence for significant mortality selection or healthy participant effects. Also, even if the healthy participant effects were stronger among obese adults, they are not strong enough to produce a weakening association between obesity and morbidity at higher ages at the time of the survey. Therefore, neither of these selection biases explains the diminishing effect of class II/III obesity on mortality over the life course.

## Introduction

Obesity has emerged as a potential threat to future increases in life expectancy, but the extent of this threat is still being debated [1–7]. Recent studies have reported population heterogeneity in obesity’s effect on mortality, as well as an age-dependence of this effect [8–14]. Most studies find that the deleterious effect of obesity on longevity weakens with age [9, 10, 14–16]; with some studies finding no harmful effect [12, 17] or even a protective effect of obesity among older adults [11, 13]. Other recent research suggests obesity may be potentially more harmful for older adults than younger adults [18], but this may be due to model misspecification and result misinterpretation [19, 20].

Prior studies on this topic mostly rely on survey data with linked mortality files to estimate the hazard ratio associated with obesity, making their findings potentially vulnerable to two sources of selection bias: mortality selection and healthy participant effects. Mortality selection refers to survival of the fittest over time. This process eliminates individuals who are frailer or have severe diseases early on in the life course, and leaves those who are more robust to survive to old age [21]. This selection process may operate more strongly among obese adults than among non-obese adults, as obese adults may be at greater risk of premature death from obesity-related diseases and conditions. Thus, a smaller proportion of frail individuals in the obese group might survive to older ages, compared to the non-obese group. This selection process would then weaken the observed harmful effect of obesity on mortality at older ages [14]. Such mortality selection bias is especially salient for survival analysis using time-specific hazard ratios [21], or age-specific hazard ratios in this case.

The healthy participant effect refers to the fact that survey respondents have to be healthy enough to participate in the survey, and is especially salient in surveys that exclude people who are institutionalized or hospitalized. Among older adults, the healthy participant effect may be stronger for obese as opposed to non-obese people, with the former being more likely to suffer from chronic illnesses or to be institutionalized due to poor health. This disparity may cause survey data to include substantially higher proportions of healthy older adults in the obese group, as compared to the non-obese group. This mechanism would then potentially lead to estimating a diminishing effect of obesity on mortality at older ages [18].

To accurately capture age variation in the obesity-mortality relationship, we need to adjust for these two selection biases. In this study, we used the U.S. National Health and Nutrition Examination Survey 1988–2010 with linked mortality files covering the period 1988–2011, and investigated these two sources of selection bias step by step. Age of death or censoring (i.e., attained age) was used as the time metric to model the baseline hazard function [22]. We accounted for left truncation by starting respondents’ exposure to the risk of death at their age at the time of the baseline survey [23]. We used Cox models with grouped age-dependence of obesity to test whether there are differential mortality selection effects and healthy participant effects across different body mass index (BMI) groups; and to test how these two sources of selection bias may affect estimates of the obesity-mortality link at older ages. We also tackled the issue of mortality selection by fitting a complementary log-log discrete time hazard model, adjusted for a normal distribution of frailty [24]; and using accelerated failure-time regression models, which provided relatively stable estimates under a variety of frailty assumptions [25, 26]. Next, we considered healthy participant effects, which are a form of sample selection bias. If the healthy participant effect were not random across various BMI groups, the existent econometrics and statistics literature have not yet developed appropriate methods to completely eliminate this type of sample selection bias. Therefore, we attempted to indirectly test for differential healthy participant effects by examining variation in the relationship between obesity and morbidity across age at the time of the survey. We will use a list of chronic conditions (e.g., diabetes, stroke and cancer) to measure morbidity. If differential healthy participant effects were the main reason for a weakening link between obesity and mortality at older ages [18], then the obesity-morbidity link would be weaker for people who are older at the time of the survey.

## Methods

### Data and participants

We used data from the National Health and Nutrition Examination Survey (NHANES) 1988–2010 with linked mortality files covering the period 1988–2011 (http://www.cdc.gov/nchs/nhanes.htm). The NHANES collected information about health and diet from a nationally representative sample of the noninstitutionalized civilian U.S. population, with an oversample of older adults and racial minorities. Data were collected from household interviews as well as physical examinations and laboratory tests performed in a mobile examination center. Detailed descriptions of the NHANES design, procedures, and methodologies are published elsewhere [27, 28]. We combined NHANES III data, collected between 1988 and 1994, with the data collected in six continuous waves of the NHANES, conducted from 1999 to 2010. The follow-up mortality data tracked the mortality status of respondents from the date of the survey through December 31, 2011. Mortality ascertainment was based on results from a probabilistic match between the NHANES data and the National Death Index (NDI) death certificate records. Ethics approval was not required because the analysis was performed on secondary data, which did not include personal identifiers. All analyses were adjusted for sample weights and the stratification and clustering of the sample design, except when specifically mentioned otherwise. We used listwise deletion to handle the missing data. The final sample was composed of 34,253 individuals aged 25 to 84 at the time of the survey, with 5,673 deaths occurring between ages 25.8 and 102.8 years (Table 1).

**Table 1:** Weighted Descriptive Statistics of Participants in the National Health and Nutrition Examination Survey, United States, 1988–2010.

	NHANES III,		NHANES III,		NHANES		NHANES		NHANES		NHANES		NHANES		NHANES	
	1988–1991		1991–1994		1999–2000		2001–2002		2003–2004		2005–2006		2007–2008		2009–2010	
	Mean (SD)	No.	Mean (SD)	No.	Mean (SD)	No.	Mean (SD)	No.	Mean (SD)	No.	Mean (SD)	No.	Mean (SD)	No.	Mean (SD)	No.
Sample size		5341		6348		2844		3668		3664		3749		4214		4425
No. of deaths		1990		1862		508		445		413		230		162		63
Normal weight		2072		2149		815		1067		1040		1011		1082		1073
Overweight		1967		2301		1058		1421		1344		1335		1475		1510
Class I obese		869		1174		552		697		786		803		928		1008
Class II/III obese		433		724		419		483		494		600		729		834
Demographics																
Age at survey, years	51 (17)		49 (17)		51 (17)		50 (16)		52 (17)		50 (17)		51 (15)		50 (15)	
Birth year	1938.7 (17)		1943.3 (17)		1948.7 (17)		1952.1 (16)		1952.3 (17)		1956.4 (17)		1957.5 (15)		1960.2 (15)	
Male		2737		2822		1364		1787		1796		1830		2081		2161
Non-Hispanic White		2654		2544		1308		1951		1944		1880		1967		2138
Non-Hispanic Black		1371		1850		534		695		709		874		887		832
Hispanic		1161		1651		913		912		867		843		1189		1226
Other race/ethnicity		155		303		89		110		144		152		171		229
U.S. born		4502		5022		2073		2925		2907		2941		3165		3217
Socioeconomic factors																
Income	26204		23440		27019		29690		26274		28113		25756		25780	
	(15518)		(14232)		(18083)		(17634)		(16577)		(15516)		(14896)		(14795)	
Less than high school		2058		2447		1065		1045		1045		983		1262		1206
High school		1632		2010		641		836		899		889		1004		1003
More than high school		1651		1891		1138		1787		1720		1877		1948		2216
Married		3480		3890		1753		2345		2190		2238		2449		2507
Partner		130		256		120		190		212		283		272		336
Separated		176		264		121		133		99		120		171		167
Widowed		549		612		252		276		332		281		273		278
Never married		554		759		314		374		438		421		527		574
Divorced		452		567		284		350		393		406		522		563
Health insurance		4792		5191		2289		3024		2998		2990		3254		3371
Health and behavioral factors																
Current smoker		1437		1546		577		810		823		800		961		978
Former smoker		1570		1642		802		1006		1033		997		1076		1131
Never smoked		2334		3160		1465		1852		1808		1952		2177		2316
Number of chronic illnesses	1 (0–2)*		1 (0–2)*		1 (0–1)*		1 (0–1)*		1 (0–2)*		1 (0–1)*		1 (0–2)*		1 (0–2)*	

^*^ Denotes median (Interquartile Range)

### Predictors of mortality

Body mass index (BMI) categories were defined using the World Health Organization guidelines, and included normal weight (BMI of 18.5–24.9 kg/m^2^), overweight (BMI of 25–29.9 kg/m^2^), class I obese (BMI of 30–34.9 kg/m^2^), and class II/III obese (BMI ≥35 kg/m^2^). Underweight, or BMI below 18.5, is linked to wasting diseases at old age. Therefore, we have excluded 522 respondents in the underweight category from the sample. Consequently, this study compared survival time between obese adults and adults in the normal weight or overweight categories. In our analysis and many other studies as well, overweight is not found to bring extra mortality risk compared to normal weight. Separating overweight from normal weight does not change the results. In order to be consistent with some prior studies in the same topic [18], we decided to combine the normal and overweight groups into a single reference category. Demographic variables in our analysis included age at the time of the survey, sex (male = 1, female = 0), race/ethnicity (non-Hispanic White, non-Hispanic Black, Hispanic, or all others), and country of birth (U.S. = 1, any other country = 0). Ten-year birth cohorts were coded from 1 (1900–1909) to 9 (1980–1989). Socioeconomic factors included inflation-adjusted family income, educational attainment (less than high school, high school diploma, more than high school), marital status (married, cohabiting, separated, widowed, never married, divorced), and health insurance status (has insurance = 1, no insurance = 0). Health and behavioral factors included smoking status (never smoked, former smoker, and current smoker), and the number of self-reported chronic conditions (angina, arthritis, asthma, bronchitis, diabetes, emphysema, heart attack, heart failure, cancer, stroke, hip fracture, spine fracture, wrist fracture, and osteoporosis). Smoking status were controlled to mitigate the confounding problem because smoking status is negatively correlated with body mass index and positively correlated with mortality. Failure to properly control for smoking status could result in underestimating the mortality risks of obesity. The chronic conditions were controlled to mitigate the reverse causation of disease-induced weight loss that may lead to underestimation of the morality risks associated with obesity.

### Statistical analysis

The time metric in all survival models was attained age: that is, the age of death or censoring. To account for left truncation, the individual-level data were reshaped into a person-year file describing respondents’ cumulative survival time from their age at the baseline survey until the age at which they died or were censored. In these person-year data, attained age was categorized into 10-year groups (25–34, 35–44, 45–54, 55–64, 65–74, 75–84, and 85+), and these categories were coded 1 through 7. This age variable is different from the time-scale variable (i.e., attained age which is a continuous variable), and is required to assess the interaction between age and obesity (or the age-dependent association between obesity and mortality.) For sensitivity analysis, age was also categorized into 5-year groups which do not change the main findings.

Model 1 was a Cox model for the whole sample, and included grouped age-dependence of obesity:

$$\text{log}h_{i}\left(t\right)=\text{log}h_{0}\left(t\right)+\gamma_{1}obeseI_{i}+\gamma_{2}obeseII_{i}+\gamma_{3}obeseI_{i}\times Age_{i}+\gamma_{4}obeseII_{i}\times Age_{i}+\sum_{j}\beta_{j}x_{ij},$$

where *h*_*i*_(*t*) is the hazard function for individual *i*; *h*_0_(*t*) is the baseline hazard function; *t* is attained age; *Age*_*i*_ represents the seven 10-year age groups; *obeseI* refers to class I obesity and *obeseII* refers to class II/III obesity (with the reference BMI group being normal or overweight); and *x*_*ij*_ are *j* control variables, including race, gender, country of birth, marital status, education, income, health insurance, smoking status, number of chronic conditions and survey year. The main variables of interest here are *obeseI* × *Age* and *obeseII* × *Age*. If the effects of obesity on mortality were to decline with age, *γ*_3_ and *γ*_4_ would be negative, and the corresponding hazard ratios would be smaller than 1.

Models 2–4 were Cox models stratified by BMI status (normal weight and overweight; class I obese; and class II/III obese, respectively), as specified below:

$$\text{log}h_{i}\left(t\right)=\text{log}h_{0}\left(t\right)+\sum_{j}\beta_{j}x_{ij}+\beta_{cy}birthcohort_{i}\times surveyyear_{i}+\beta_{c}birthcohort_{i}+\beta_{y}surveyyear_{i}.$$

The main variable of interest here is the interaction between birth cohort and survey year. If there were a selection bias due to the healthy participant effect or mortality selection, individuals in the same cohort who were interviewed in a later period (i.e., at older ages) would have a lower mortality risk than those who were interviewed in an earlier period (i.e., at younger ages). Therefore, if either of the two effects exists, we would observe a negative *β*_*cy*_ within each BMI category. If either of the two effects were more pronounced among obese adults, we would observe a stronger negative effect *β*_*cy*_ in the obese I and obese II/III groups, as compared to the normal weight and overweight group.

Model 5 was a Cox model for the whole sample, adjusted for both mortality selection and sample selection bias:

$$\text{log}h_{i}\left(t\right)=\text{log}h_{0}\left(t\right)+\gamma_{1}obeseI_{i}+\gamma_{2}obeseII_{i}+\gamma_{3}obeseI_{i}\times Age_{i}+\gamma_{4}obeseII_{i}\times Age_{i}+\underset{j}{\sum }\beta_{j}x_{ij}+\beta_{cy}birthcohort_{i}\times surveyyear_{i}+\beta_{c}birthcohort_{i}+\beta_{y}surveyyear_{i}$$

If mortality selection or healthy participant effects accounted for the diminishing effect of obesity on mortality, *γ*_3_ and *γ*_4_ would be substantially reduced here, relative to the estimates of these coefficients in Model 1.

Model 6 was a complementary log-log discrete time hazard model, adjusted for a normal distribution of frailty:

$$\text{log}\left[-\text{log}\left(1-P_{ik}\right)\right]=\alpha \text{log}k+\gamma_{1}obeseI_{ik}+\gamma_{2}obeseII_{ik}+\gamma_{3}obeseI_{ik}\times k+\gamma_{4}obeseII_{ik}\times k+\sum_{j}\beta_{j}x_{ikj}+u_{i},$$

where *k* represents the seven 10-year age groups; *P*_*ik*_ is the probability that individual *i* in age group *k* dies; and *u*_*i*_ is the unobserved frailty term, assumed to be normally distributed. If there were significant differences in mortality selection among BMI groups, the variance of the frailty distribution would be significant and *γ*_3_ and *γ*_4_ would be substantially reduced, relative to the estimates of these coefficients in Model 1.

Hazard ratios, however, may be sensitive to the choice of the parametric form of the frailty distribution [26, 29]. Therefore, we used a series of accelerated failure-time regression models (AFT) in Models 7–9:

$$\text{log}\left(T_{i}\right)=\propto_{0}+\gamma_{1}obeseI_{i}+\gamma_{2}obeseII_{i}+\gamma_{3}obeseI_{i}\times Age_{i}+\gamma_{4}obeseII_{i}\times Age_{i}+\sum_{j}\beta_{j}x_{ij}+\varepsilon_{i},$$

where *T*_*i*_ denotes the survival time for the *i*th individual; *ε*_*i*_ is a measure of residual variability in the survival times and is assumed to have a log-Weibull distribution (Model 7), log-gamma distribution (Model 8), or normal distribution (Model 9), meaning the distribution of *T* would be Weibull, gamma, or log-normal, respectively. One advantage of the AFT over the hazard model is that the AFT is much less affected by different assumptions about the frailty distribution: when a heterogeneity or frailty term is added to AFT models, it only contributes to the dispersion, leaving regression coefficients unchanged [25, 26]. If obesity were detrimental to survival, *γ*_1_ and *γ*_2_ would be negative, and the corresponding time ratios would be smaller than 1. If the effects of obesity on mortality were to decline with age, *γ*_3_ and *γ*_4_ would be positive, and the corresponding time ratios would be greater than 1.

In order to completely remove the confounding problem due to smoking status and reverse causation due to disease-induced weight loss, we also constrained our analyses to people without smoking history or preexisting chronic conditions. Those results are presented in Supporting Information Tables.

In order to further test the healthy participant effect, we investigated whether the effect of obesity on morbidity (measured by the number of chronic illnesses) is different across age at the time of the baseline survey. Here, we used the original individual-level data and fitted an ordinary least squares regression model. If the old-age decline in mortality risk associated with obesity were a result of a more pronounced healthy participant effect in obese groups [18], then the correlation between obesity and morbidity at the time of the survey would be weaker among older respondents.

## Results

Table 2 presents adjusted hazard ratios of obesity from weighted Cox models. Model 1, which uses “normal weight and overweight” as the reference BMI category, shows that every ten year increase in age decreases class II/III obesity-related hazard by 15%. The effect of class I obesity is significant which, however, does not change over the life course.

**Table 2:** Adjusted Hazard Ratios from Weighted Cox Model, NHANES III-NHANES 2009–2010, United States.

	Model 1 ^a^		Model 2^b^		Model 3^b^		Model 4^b^	
	(age as time metric)		(normal weight + overweight)		(class I obese)		(class II/III obese)	
	HR	95% CI	HR	95% CI	HR	95% CI	HR	95% CI
Reference BMI (18.5–29.9)								
Class I obese (30.0–34.9)	1.59	1.05, 2.41						
Class II/III obese (35.0+)	3.41	2.08, 5.60						
Class I obese * Age	0.94	0.87, 1.01						
Class II/III obese * Age	0.85	0.77, 0.92						
Birth cohort * Survey year			1.02	1.00, 1.04	1.03	0.99, 1.07	1.03	0.98, 1.08

Abbreviations: BMI, body mass index; CI, confidence interval; HR, hazard ratio; NHANES, National Health and Nutrition Examination Survey.

^a^ Adjusted for race, gender, country of birth, marital status, education, income, health insurance, smoking status, chronic conditions and survey year.

^b^ Adjusted for race, gender, country of birth, marital status, education, income, health insurance, smoking status, chronic conditions, survey year and birth cohort.

The next three models present adjusted hazard ratios of interactions between birth cohort and survey year, obtained from weighted Cox models fitted separately for each BMI group. These results suggest that, in the same cohort, subjects who were interviewed in later periods (i.e., at older ages) were not significantly less likely to die than those who were interviewed in earlier periods (i.e., at younger ages). This finding is consistent across the three BMI groups (normal weight and overweight; class I obese; and class II/III obese), and suggests no existence of significant healthy participant or mortality selection effects. Age at baseline survey was not controlled in these models because in order to account for left-truncation problem, the converted person-year data already started with the respondents’ age at the baseline survey until the age at which they died or were censored. Adding age at baseline survey to these models did not change the findings. In fact, age at baseline survey was not significant. We also replaced the interaction between birth cohort and survey year with the interaction between birth cohort and age at baseline survey. These new interaction terms were not significant either which further suggests no significant healthy participant or mortality selection effects among these BMI groups.

Model 5, presented in Table 3, adjusts for both healthy participant and mortality selection effects by adding interactions between birth cohort and survey year to the covariates from Model 1. The results remain almost the same when compared to Model 1: the positive association between class II/III obesity and mortality risk is significantly smaller at older ages. Model 6 replicates this finding with an adjustment for a normal distribution of frailty. A likelihood ratio test of the frailty distribution’s variance suggests that there is no significant unobserved frailty. AFT Models 7–9, presented in Table 4, suggest that class II/III obesity significantly reduces survival time by about 17–19%, but this detrimental effect weakens over the life course.

These findings further refute the hypothesis that differential mortality selection across BMI groups leads to weaker class II/III obesity effects on mortality risk later in the life course.

**Table 3:** Adjusted Hazard Ratios of Obesity Relative to Normal Weight and Overweight across the Life Course, NHANES III-NHANES 2009–2010, United States.

	Model 5 ^a^		Model 6 ^b^	
	(adjusted for selection effects)		(adjusted for normal distribution of frailty)	
	HR	95% CI	HR	95% CI
Reference BMI (18.5–29.9)				
Class I obese (30.0–34.9)	1.56	1.03, 2.35	1.31	0.94, 1.84
Class II/III obese (35.0+)	3.26	2.00, 5.31	3.14	2.16, 4.57
Class I obese * Age	0.94	0.87, 1.01	0.95	0.89, 1.00
Class II/III obese * Age	0.85	0.78, 0.93	0.82	0.76, 0.88
Birth cohort * Survey year	1.02	1.00, 1.03		
Likelihood ratio test of the frailty distribution variance			*P =* .*480*	

Abbreviations: BMI, body mass index; CI, confidence interval; HR, hazard ratio; NHANES, National Health and Nutrition Examination Survey.

^a^ from Weighted Cox Model, adjusted for race, gender, country of birth, marital status, education, income, health insurance, smoking status, chronic conditions, survey year and birth cohort.

^b^ from Weighted Complementary Log-log Discrete Time Hazard Model, adjusted for logarithm of age, race, gender, country of birth, marital status, education, income, health insurance, smoking status, chronic conditions and survey year. This model is not weighted because complementary log-log models (xtcloglog) are not supported by the survey weights command (svy) in Stata.

**Table 4:** Adjusted Time Ratios of Obesity Relative to Normal Weight and Overweight across the Life Course from Weighted Accelerated Failure-Time Regression Model, NHANES III-NHANES 2009–2010, United States.

	Model 7 ^a^		Model 8 ^b^		Model 9 ^c^	
	TR	95% CI	TR	95% CI	TR	95% CI
Reference BMI (18.5–29.9)						
Class I obese (30.0–34.9)	0.96	0.89, 1.03	0.95	0.89, 1.02	0.94	0.86, 1.03
Class II/III obese (35.0+)	0.81	0.75, 0.88	0.82	0.76, 0.89	0.83	0.75, 0.92
Class I obese * Age	1.01	0.99, 1.02	1.01	1.00, 1.02	1.01	0.99, 1.03
Class II/III obese * Age	1.03	1.02, 1.05	1.03	1.02, 1.04	1.03	1.01, 1.06

Abbreviations: BMI, body mass index; CI, confidence interval; TR, time ratio; NHANES, National Health and Nutrition Examination Survey.

^a^ Assuming Weibull distribution of T, adjusted for race, gender, country of birth, marital status, education, income, health insurance, smoking status, chronic conditions and survey year.

^b^ Assuming gamma distribution of T, adjusted for race, gender, country of birth, marital status, education, income, health insurance, smoking status, chronic conditions and survey year.

^c^ Assuming log-normal distribution of T, adjusted for race, gender, country of birth, marital status, education, income, health insurance, smoking status, chronic conditions and survey year.

S1–S6 Tables present the findings among people without smoking history or preexisting chronic conditions. Overall, these findings are very similar to those in Tables 2–4. This suggests neither the confounding problem due to smoking status nor reverse causation due to diseases-induced weight loss are the sources for the declining effect of class II/III obesity on mortality over life course.

**Fig 1:**
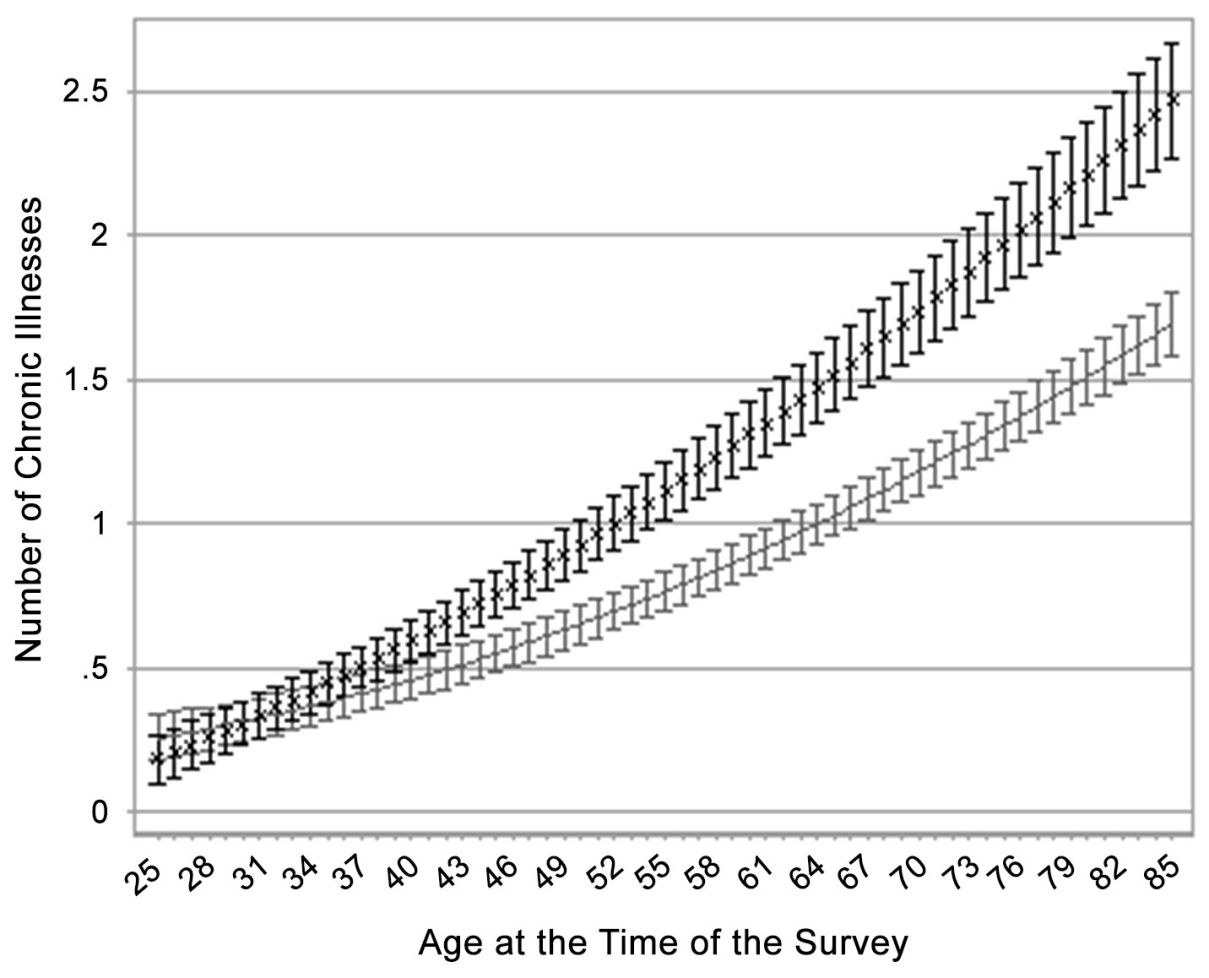
Estimated number of chronic illnesses in two body mass index groups across age at survey from weighted OLS (ordinary least squares) regression model. Data are from the U.S. National Health and Nutrition Examination Survey 1988–2010. Model was adjusted for race, gender, country of birth, marital status, education, income, health insurance, smoking status, and survey year. The body mass index groups are as follows: solid line, normal weight and overweight; ×, class II/III obese; and I, 95% confidence bands.

Fig 1 illustrates the predicted number of chronic illnesses across age at the time of the survey, stratified by BMI category. Fig 1 is derived from a weighted OLS regression of the number of chronic illnesses on BMI category, age at survey, age at survey squared, the interaction between BMI category and age at survey, and additional individual covariates (full results from this model are presented in S7 Table). The gap in the number of chronic illnesses between class II/III obese adults and adults in the normal or overweight category widens significantly at older ages at time of survey (gaps in this outcome among normal or overweight, class I obese, and class II/III obese respondents are summarized in S1 Fig). This finding suggests that even if the healthy participant effect were stronger among the obese group (an argument not supported by Table 2), this effect would not be strong enough to weaken the link between obesity and morbidity at older ages. Therefore, differential healthy participant effects across BMI categories probably do not explain the declining mortality effect of class II/III obesity over the life course.

## Discussion

Although several studies have reported that the adverse effect of obesity on mortality risk decreases over the life course [9, 10, 14–16], this phenomenon may be caused by more pronounced mortality selection and healthy participant effects among obese adults. If there were a selection bias due to the healthy participant effect or mortality selection, individuals in the same cohort who were interviewed in a later period (i.e., at older ages) would have a lower mortality risk than those who were interviewed in an earlier period (i.e., at younger ages) although this phenomenon could be also partly caused by the secular declines in the obesity-mortality association over time [30]. If these selection effects were more pronounced among obese adults, we should find this lower mortality risk would be more salient among obese than non-obese individuals. Using NHANES data and a series of Cox models, we found no evidence that mortality selection or healthy participant effects were significantly more pronounced among obese adults, as compared to normal- and overweight adults. We also used complementary log-log models, adjusted for a normal distribution of frailty, to test for mortality selection effects, and found no significant difference in unobserved frailty across different BMI groups. As estimates of the hazard ratios suffer from bias induced by the mortality selection (or unobserved frailty) problem [21, 31], we also used a series of accelerated failure-time regression models, which are much less affected by different assumptions about the frailty distribution [25, 26], and achieved similar results. Last, we did not find the gap in the number of chronic illnesses between class II/III obese adults and adults in the normal or overweight category significantly narrows at older ages at time of survey as predicted by healthy participants effects. We replicated all the analyses using National Health Interview Survey data from the years 1986–2004, with linked mortality files covering the period 1986–2006, and obtained results consistent with our main findings (S8 and S9 Tables). Therefore, neither source of selection bias can explain why the effect of class II/III obesity on mortality declines with age. Moreover, additional analyses presented in S1–S6 Tables suggest that neither the confounding problem due to smoking status nor reverse causation due to diseases-induced weight loss contributes to this pattern. It deserves clarification that while the associations are of smaller magnitude among older people on the relative scale, they are greater on the absolute scale. Because mortality is so much greater in older people than younger people, the absolute number of deaths associated with obesity is greater in older people even though the hazard ratio is lower.

There are three other possible explanations for the weakening link between obesity and mortality over the life course. First, the hazard ratios of death for many behavioral factors and bio-indicators including obesity likely diminish with increasing age perhaps due to the fact that multiple body systems are failing and the excess risks of any single risk factor (even if it affects more than one body system) will likely fall due to the competing risks. Second, the accumulation of time spent obese, or the cumulative amount of body mass, may contribute to mortality risk beyond weight status at a single point in time. People who become obese at earlier ages are at significantly higher risk of death compared to those who become obese at older ages because they tend to remain so for most of their life course [32]. Modest weight gain after age 50, however, does not confer excess mortality risk [32–34]. Therefore, the accumulation of time in the obese category among people who were obese in early adulthood could increase their mortality hazard above that of people who became obese in later adulthood [32]. Moreover, people who are obese at older ages are a heterogeneous group, some of whom may have been overweight or normal weight at younger ages. The heterogeneity in population composition with regard to duration of obesity for different age groups may contribute to the age pattern of the obesity-mortality association.

Third, although obesity is associated with the onset of many chronic diseases that increase the mortality hazard in old age, being obese may actually improve survival after the onset of certain diseases [35]. The combination of these two patterns may then produce a declining effect of obesity on mortality later in the life course. One study that followed 34,132 patients who experienced an acute ischemic stroke found obese patients had lower long-term mortality rates [36]. Patients whose BMI classified them as obese (30–32.5 and >32.5 kg/m^2^) had lower hazard ratios (HR) (0.77 (95% CI: 0.63, 0.93) and 0.85 (95% CI: 0.64, 1.12), respectively) compared to the normal weight reference group (BMI in the 20–23 kg/m^2^ range) [36]. Another clinical study investigated the risks of death, nonfatal myocardial infarction, and nonfatal stroke among patients with hypertension and coronary artery disease, finding that these risks were lower among class I obese patients (adjusted HR = 0.68 (95% CI: 0.59, 0.78)) and class II/III obese patients (adjusted HR = 0.76 (95% CI: 0.65, 0.88)), compared to normal weight patients [37]. Some recent research, however, suggests that the survival advantage of being obese in populations diagnosed with a medical condition (e.g., dysglycemia) may be due to unmeasured risk factors (e.g., smoking) [38]. They found illness-associated weight loss was more prevalent among smokers than among never-smokers; and among dysglycemia patients without smoking history, the survival advantage of being obese disappeared. But it may be too early to conclude that survival advantage of being obese for other diseases is also completely due to the similar selection bias.

Differential healthy participant effects imply older adults who are obese are more likely to be hospitalized and be excluded from surveys than their non-obese peers, leading analyses of survey data to suggest a lower obesity effect on mortality at older ages. This hypothesis presumes that obese people who are hospitalized are weaker and more likely to die than non-obese people who are hospitalized; and that if we could include the hospitalized population in the sampling frame, the effect of obesity on mortality risk would not decline with age. But studies have found that obesity predicts lower in-hospital mortality [39]. One study that monitored 16,812 Intensive Care Unit (ICU) patients at a Boston hospital found that obese patients had 26% (95% CI: 0.14, 0.36) lower mortality risk 30 days after ICU admission and 43% (95% CI: 0.33, 0.51) lower mortality risk one year after ICU admission, compared to normal weight patients [40]. Another study that analyzed 108,927 hospitalizations for acute decompensated heart failure found that patients in higher BMI categories had lower in-hospital mortality after heart failure [41]. This risk decreased with higher BMI in a nearly linear fashion. Using BMI as a continuous variable, every 5 unit increase in BMI was associated with 10% (95% CI: 0.07, 0.12) lower odds of risk-adjusted mortality [41]. These studies further dispute the plausibility of a differential healthy participant effect causing the effect of obesity on mortality to diminish at older ages.

The reasons why obese people are less likely to die from some chronic illnesses than non-obese people remain unclear. Extra body weight, including both fat mass and lean tissue mass, may serve as a nutrition reservoir to help fight against chronic illness. For example, studies on patients with heart failure suggest normal weight patients may not have enough metabolic reserves to overcome the catabolic stress associated with heart failure [41]. These nutritional reserves may also shield obese elderly people from weight loss, as people tend to eat less when they get older, regardless of health status [42]. In addition, patients with heart failure have been shown to have low levels of insulinlike growth factor 1 and elevated levels of several factors that are associated with anorexia and muscle wasting, indicating cardiac cachexia and an increased risk of adverse outcomes [35]. The “normal weight” category may include a higher proportion of cachectic patients than higher BMI categories, as these patients are more susceptible to weight loss and muscle wasting.

Another explanation for why sick people may experience a protective effect from obesity in old age is that obese patients seek or receive treatment at an earlier stage of many diseases. Obese people have a readily identifiable phenotype that is believed to reflect a number of diseases, and therefore may be subjected to earlier and more regular monitoring compared to normal weight patients [37]. In addition, the elderly obese may have better nutritional intake [43], greater fitness levels [44], or less sedentary lifestyles [45] than the normal weight elderly; as well as greater bone density, predicting a lower risk of hip fracture [42]. Finally, animal studies show that increases in adipocyte size in obese organisms lead to an accumulation of M1 macrophages in adipose tissue that produce pro-inflammatory cytokines [40]. But when patients get critically ill, they may develop M2 macrophages that produce anti-inflammatory agents [40]. Obese patients may experience a protective effect during critical illness that entails a switch from M1 pro-inflammatory activation to M2 anti-inflammatory activation in the larger pool of macrophages their adipose tissue stores, compared to normal weight patients [40].

In conclusion, this study finds that the mortality hazard associated with class II/III obesity declines over the life course, and that this decline is not a result of more pronounced mortality selection or healthy participant effects among obese adults as compared to non-obese adults. Instead, this pattern may be related to the competing risks in the older ages, the duration of exposure to obesity and a possible protective effect of extra body weight against death from chronic illness in old age. Public health efforts aimed at controlling obesity should focus on earlier stages of the life course, rather than obesity among the elderly.

## Supporting Information

S1 Fig Estimated number of chronic illnesses in three body mass index groups across age at the time of the survey from weighted OLS (ordinary least squares) regression model. Data are from the U.S. National Health and Nutrition Examination Survey 1988–2010. Model was adjusted for race, gender, country of birth, marital status, education, income, health insurance, smoking status, and survey year. The body mass index groups are as follows: solid line, normal weight and overweight; open circle, class I obese; and ×, class II/III obese.
(DOCX)

S1 Table Adjusted Hazard Ratios from Weighted Cox Model among Non-Smokers, NHANES III-NHANES 2009–2010, United States. (DOCX)

S2 Table Adjusted Hazard Ratios of Obesity Relative to Normal Weight and Overweight across the Life Course among Non-Smokers, NHANES III-NHANES 2009–2010, United States. (DOCX)

S3 Table Adjusted Time Ratios of Obesity Relative to Normal Weight and Overweight across the Life Course from Weighted Accelerated Failure-Time Regression Model among Non-Smokers, NHANES III-NHANES 2009–2010, United States. (DOCX)

S4 Table Adjusted Hazard Ratios from Weighted Cox Model among People without Preexisting Chronic Conditions, NHANES III-NHANES 2009–2010, United States. (DOCX)

S5 Table Adjusted Hazard Ratios of Obesity Relative to Normal Weight and Overweight across the Life Course among People without Preexisting Chronic Conditions, NHANES III-NHANES 2009–2010, United States. (DOCX)

S6 Table Adjusted Time Ratios of Obesity Relative to Normal Weight and Overweight across the Life Course from Weighted Accelerated Failure-Time Regression Model among People without Preexisting Chronic Conditions, NHANES III-NHANES 2009–2010, United States. (DOCX)

S7 Table Adjusted Coefficients of BMI Groups on the Number of Chronic Illnesses across Age at the Time of the Survey from Ordinary Least Squares Regression, NHANES III-NHANES 2009–2010, United States. (DOCX)

S8 Table Adjusted Hazard Ratios of Obesity Relative to Normal Weight and Overweight across the Life Course from Weighted Cox Model in Adult Men, NHIS 1986–2006, United States. (DOCX)

S9 Table Adjusted Hazard Ratios of Obesity Relative to Normal Weight and Overweight across the Life Course from Weighted Cox Model in Adult Women, NHIS 1986–2006, United States. (DOCX)
