# Supplementary material for: The Body Mass Index-Mortality Link across the Life Course: Two Selection Biases and Their Effects
Source: PLoS One. 2016 Feb 3;11(2):e0148178. doi: 10.1371/journal.pone.0148178 (PMC4739746; doi:10.1371/journal.pone.0148178)

Figure S1. Estimated number of chronic illnesses in three body mass index groups across age at the time of the survey from weighted OLS (ordinary least squares) regression model. Data are from the U.S. National Health and Nutrition Examination Survey 1988-2010. Model was adjusted for race, gender, country of birth, marital status, education, income, health insurance, smoking status, and survey year. The body mass index groups are as follows: solid line, normal weight and overweight; open circle, class I obese; and ×, class II/III obese.


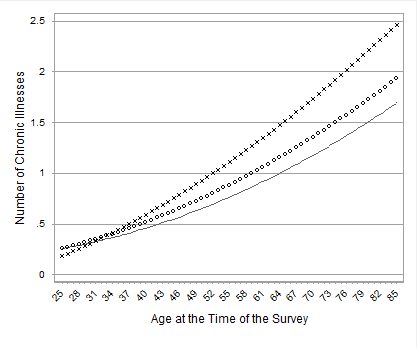

Supplement: S1 Fig — Data are from the U.S. National Health and Nutrition Examination Survey 1988–2010. Model was adjusted for race, gender, country of birth, marital status, education, income, health insurance, smoking status, and survey year. The body mass index groups are as follows: solid line, normal weight and overweight; open circle, class I obese; and ×, class II/III obese. (DOCX) [file pone.0148178.s001.docx]
