# Supplementary material for: The Body Mass Index-Mortality Link across the Life Course: Two Selection Biases and Their Effects
Source: PLoS One. 2016 Feb 3;11(2):e0148178. doi: 10.1371/journal.pone.0148178 (PMC4739746; doi:10.1371/journal.pone.0148178)
Supplement: S1 Table — (DOCX) [file pone.0148178.s002.docx]

Table S1**.** Adjusted Hazard Ratios from Weighted Cox Model among Non-Smokers, NHANES III-NHANES 2009-2010, United States

|  | **Model 1 ^a^**  **(age as time metric)** | | **Model 2^b^**  **(normal weight + overweight)** | | | **Model 3^b^**  **(class I obese)** | | | **Model 4^b^**  **(class II/III obese)** | |
| --- | --- | --- | --- | --- | --- | --- | --- | --- | --- | --- |
|  | **HR** | **95% CI** | **HR** | **95% CI** | **HR** | | **95% CI** | **HR** | | **95% CI** |
| Reference BMI (18.5-29.9) |  |  |  |  |  | |  |  | |  |
| Class I obese (30.0-34.9) | 1.66 | 0.78, 3.51 |  |  |  | |  |  | |  |
| Class II/III obese (35.0+) | 3.39 | 1.58, 7.31 |  |  |  | |  |  | |  |
| Class I obese * Age | 0.94 | 0.83, 1.07 |  |  |  | |  |  | |  |
| Class II/III obese * Age | 0.86 | 0.76, 0.96 |  |  |  | |  |  | |  |
| Birth cohort * Survey year |  |  | 1.00 | 0.97, 1.03 | 1.07 | | 1.02, 1.12 | 0.98 | | 0.92, 1.05 |

Abbreviations: BMI, body mass index; CI, confidence interval; HR, hazard ratio; NHANES, National Health and Nutrition Examination Survey.

^a^ Adjusted for race, gender, country of birth, marital status, education, income, health insurance, chronic conditions and survey year.

^b^ Adjusted for race, gender, country of birth, marital status, education, income, health insurance, chronic conditions, survey year and birth cohort.
