# Supplementary material for: The Body Mass Index-Mortality Link across the Life Course: Two Selection Biases and Their Effects
Source: PLoS One. 2016 Feb 3;11(2):e0148178. doi: 10.1371/journal.pone.0148178 (PMC4739746; doi:10.1371/journal.pone.0148178)
Supplement: S2 Table — (DOCX) [file pone.0148178.s003.docx]

Table S2**.** Adjusted Hazard Ratios of Obesity Relative to Normal Weight and Overweight across the Life Course among Non-Smokers, NHANES III-NHANES 2009-2010, United States

|  | **Model 5 ^a^**  **(adjusted for selection effects)** | | **Model 6 ^b^**  **(adjusted for normal distribution of frailty)** | | |
| --- | --- | --- | --- | --- | --- |
|  | **HR** | **95% CI** | **HR** | **95% CI** |  |
| Reference BMI (18.5-29.9) |  |  |  |  |  |
| Class I obese (30.0-34.9) | 1.65 | 0.78, 3.49 | 2.09 | 1.19, 3.70 |  |
| Class II/III obese (35.0+) | 3.40 | 1.56, 7.43 | 4.88 | 2.70, 8.81 |  |
| Class I obese * Age | 0.94 | 0.83, 1.07 | 0.87 | 0.79, 0.96 |  |
| Class II/III obese * Age | 0.85 | 0.75, 0.96 | 0.76 | 0.68, 0.84 |  |
| Birth cohort * Survey year | 1.01 | 0.99, 1.04 |  |  |  |
| Likelihood ratio test of the frailty distribution variance |  | | *P = .488* | | |

Abbreviations: BMI, body mass index; CI, confidence interval; HR, hazard ratio; NHANES, National Health and Nutrition Examination Survey.

^a^ from Weighted Cox Model, adjusted for race, gender, country of birth, marital status, education, income, health insurance, chronic conditions, survey year and birth cohort.

^b^ from Weighted Complementary Log-log Discrete Time Hazard Model, adjusted for logarithm of age, race, gender, country of birth, marital status, education, income, health insurance, chronic conditions and survey year. This model is not weighted because complementary log-log models (xtcloglog) are not supported by the survey weights command (svy) in Stata.
