# Supplementary material for: The Body Mass Index-Mortality Link across the Life Course: Two Selection Biases and Their Effects
Source: PLoS One. 2016 Feb 3;11(2):e0148178. doi: 10.1371/journal.pone.0148178 (PMC4739746; doi:10.1371/journal.pone.0148178)
Supplement: S4 Table — (DOCX) [file pone.0148178.s005.docx]

Table S4**.** Adjusted Hazard Ratios from Weighted Cox Model among People without Preexisting Chronic Conditions, NHANES III-NHANES 2009-2010, United States

|  | **Model 1 ^a^**  **(age as time metric)** | | **Model 2^b^**  **(normal weight + overweight)** | | | **Model 3^b^**  **(class I obese)** | | | **Model 4^b^**  **(class II/III obese)** | |
| --- | --- | --- | --- | --- | --- | --- | --- | --- | --- | --- |
|  | **HR** | **95% CI** | **HR** | **95% CI** | **HR** | | **95% CI** | **HR** | | **95% CI** |
| Reference BMI (18.5-29.9) |  |  |  |  |  | |  |  | |  |
| Class I obese (30.0-34.9) | 1.81 | 1.05, 3.12 |  |  |  | |  |  | |  |
| Class II/III obese (35.0+) | 2.70 | 1.52, 4.81 |  |  |  | |  |  | |  |
| Class I obese * Age | 0.91 | 0.82, 1.01 |  |  |  | |  |  | |  |
| Class II/III obese * Age | 0.89 | 0.79, 0.99 |  |  |  | |  |  | |  |
| Birth cohort * Survey year |  |  | 1.01 | 0.99, 1.04 | 1.03 | | 0.98, 1.08 | 1.02 | | 0.95, 1.10 |

Abbreviations: BMI, body mass index; CI, confidence interval; HR, hazard ratio; NHANES, National Health and Nutrition Examination Survey.

^a^ Adjusted for race, gender, country of birth, marital status, education, income, health insurance, smoking status and survey year.

^b^ Adjusted for race, gender, country of birth, marital status, education, income, health insurance, smoking status, survey year and birth cohort.
