# Supplementary material for: The Body Mass Index-Mortality Link across the Life Course: Two Selection Biases and Their Effects
Source: PLoS One. 2016 Feb 3;11(2):e0148178. doi: 10.1371/journal.pone.0148178 (PMC4739746; doi:10.1371/journal.pone.0148178)
Supplement: S6 Table — (DOCX) [file pone.0148178.s007.docx]

Table S6**.** Adjusted Time Ratios of Obesity Relative to Normal Weight and Overweight across the Life Course from Weighted Accelerated Failure-Time Regression Model among People without Preexisting Chronic Conditions, NHANES III-NHANES 2009-2010, United States

|  | **Model 7 ^a^** | | **Model 8 ^b^** | | | **Model 9 ^c^** | | |
| --- | --- | --- | --- | --- | --- | --- | --- | --- |
|  | **TR** | **95% CI** | **TR** | **95% CI** | **TR** | | **95% CI** |  |
| Reference BMI (18.5-29.9) |  |  |  |  |  | |  |  |
| Class I obese (30.0-34.9) | 0.93 | 0.85, 1.02 | 0.93 | 0.85, 1.01 | 0.93 | | 0.82, 1.04 |  |
| Class II/III obese (35.0+) | 0.84 | 0.76, 0.93 | 0.85 | 0.78, 0.93 | 0.89 | | 0.80, 0.99 |  |
| Class I obese * Age | 1.01 | 0.99, 1.03 | 1.01 | 1.00, 1.03 | 1.01 | | 0.99, 1.04 |  |
| Class II/III obese * Age | 1.03 | 1.01, 1.05 | 1.02 | 1.01, 1.04 | 1.02 | | 0.99, 1.04 |  |

Abbreviations: BMI, body mass index; CI, confidence interval; TR, time ratio; NHANES, National Health and Nutrition Examination Survey.

^a^ Assuming Weibull distribution of T, adjusted for race, gender, country of birth, marital status, education, income, health insurance, smoking status and survey year.

^b^ Assuming gamma distribution of T, adjusted for race, gender, country of birth, marital status, education, income, health insurance, smoking status and survey year.

^c^ Assuming log-normal distribution of T, adjusted for race, gender, country of birth, marital status, education, income, health insurance, smoking status and survey year.
