# Supplementary material for: The Body Mass Index-Mortality Link across the Life Course: Two Selection Biases and Their Effects
Source: PLoS One. 2016 Feb 3;11(2):e0148178. doi: 10.1371/journal.pone.0148178 (PMC4739746; doi:10.1371/journal.pone.0148178)
Supplement: S7 Table — (DOCX) [file pone.0148178.s008.docx]

Table S7. Adjusted Coefficients of BMI Groups on the Number of Chronic Illnesses across Age at the Time of the Survey from Ordinary Least Squares Regression, NHANES III-NHANES 2009-2010, United States

|  | Coefficient | 95% CI | |
| --- | --- | --- | --- |
| Reference BMI (18.5-29.9) |  |  |  |
| Class I obese | -0.001 | -0.073 | 0.071 |
| Class II/III obese | -0.078 | -0.154 | -0.002 |
| Age at survey | 0.010 | 0.006 | 0.013 |
| Age at survey squared | 0.000 | 0.000 | 0.000 |
| Class I obese *Age at survey | 0.004 | 0.001 | 0.007 |
| Class II/III obese *Age at survey | 0.014 | 0.011 | 0.018 |

Abbreviations: BMI, body mass index; CI, confidence interval; NHANES, National Health and Nutrition Examination Survey.

^a^ Adjusted for race, gender, country of birth, marital status, education, income, health insurance, smoking status, and survey year.
