# Supplementary material for: The Body Mass Index-Mortality Link across the Life Course: Two Selection Biases and Their Effects
Source: PLoS One. 2016 Feb 3;11(2):e0148178. doi: 10.1371/journal.pone.0148178 (PMC4739746; doi:10.1371/journal.pone.0148178)
Supplement: S8 Table — (DOCX) [file pone.0148178.s009.docx]

Table S8. Adjusted Hazard Ratios of Obesity Relative to Normal Weight and Overweight across the Life Course from Weighted Cox Model in Adult Men, NHIS 1986-2006, United States

|  | Model 1  ^a^  (age as time metric) | | Model 2  ^b^  (normal weight + overweight) | | Model 3  ^b^  (class I obese) | | Model 4  ^b^  (class II/III obese) | | Model 5 ^b^  (adjusted for selection effects) | |
| --- | --- | --- | --- | --- | --- | --- | --- | --- | --- | --- |
|  | HR | 95% CI | HR | 95% CI | HR | 95% CI | HR | 95% CI | HR | 95% CI |
| Reference BMI (18.5-29.9) |  |  |  |  |  |  |  |  |  |  |
| Class I obese (30.0-34.9) | 1.32 | 1.21, 1.44 |  |  |  |  |  |  | 1.31 | 1.20, 1.43 |
| Class II/III obese (35.0+) | 2.87 | 2.50, 3.30 |  |  |  |  |  |  | 2.86 | 2.48, 3.29 |
| Class I obese * Age | .97 | .95, .98 |  |  |  |  |  |  | .97 | .95, .99 |
| Class II/III obese * Age | .88 | .85, .90 |  |  |  |  |  |  | .88 | .85, .91 |
| Birth cohort * Survey year |  |  | 1.00 | 1.00, 1.00 | 1.00 | 1.00, 1.00 | 1.00 | 1.00, 1.00 | 1.00 | 1.00, 1.00 |

Abbreviations: BMI, body mass index; CI, confidence interval; HR, hazard ratio; NHIS, National Health Interview Survey.

^a^ Adjusted for race/ethnicity, marital status, education, income, region of residence, and survey year.

^b^ Adjusted for race/ethnicity, marital status, education, income, region of residence, survey year and birth cohort.
