# Supplementary material for: The Body Mass Index-Mortality Link across the Life Course: Two Selection Biases and Their Effects
Source: PLoS One. 2016 Feb 3;11(2):e0148178. doi: 10.1371/journal.pone.0148178 (PMC4739746; doi:10.1371/journal.pone.0148178)
Supplement: S9 Table — (DOCX) [file pone.0148178.s010.docx]

Table S9. Adjusted Hazard Ratios of Obesity Relative to Normal Weight and Overweight across the Life Course from Weighted Cox Model in Adult Women, NHIS 1986-2006, United States

|  | Model 1  ^a^  (age as time metric) | | Model 2  ^b^  (normal weight + overweight) | | Model 3  ^b^  (class I obese) | | Model 4  ^b^  (class II/III obese) | | Model 5 ^b^  (adjusted for selection effects) | |
| --- | --- | --- | --- | --- | --- | --- | --- | --- | --- | --- |
|  | HR | 95% CI | HR | 95% CI | HR | 95% CI | HR | 95% CI | HR | 95% CI |
| Reference BMI (18.5-29.9) |  |  |  |  |  |  |  |  |  |  |
| Class I obese (30.0-34.9) | 1.59 | 1.43, 1.76 |  |  |  |  |  |  | 1.60 | 1.44, 1.77 |
| Class II/III obese (35.0+) | 2.57 | 2.29, 2.89 |  |  |  |  |  |  | 2.60 | 2.31, 2.92 |
| Class I obese * Age | .94 | .92, .95 |  |  |  |  |  |  | .94 | .92, .95 |
| Class II/III obese * Age | .91 | .89, .93 |  |  |  |  |  |  | .90 | .88, .92 |
| Birth cohort * Survey year |  |  | 1.00 | 1.00, 1.00 | 1.00 | 1.00, 1.00 | 1.00 | 1.00, 1.00 | 1.00 | 1.00, 1.00 |

Abbreviations: BMI, body mass index; CI, confidence interval; HR, hazard ratio; NHIS, National Health Interview Survey.

^a^ Adjusted for race/ethnicity, marital status, education, income, region of residence, and survey year.

^b^ Adjusted for race/ethnicity, marital status, education, income, region of residence, survey year and birth cohort.
